# Protective Effects of Xanthorrhizol-Rich Extracts Against PM-Induced Skin Damage in Human Keratinocytes and 3D-Reconstructed Skin Models

**DOI:** 10.3390/ph18060808

**Published:** 2025-05-28

**Authors:** Haneul Kang, Eun-Ji Ko, Dahye Lee, Junhui Kang, Jae-Kwan Hwang, Eunsoo Kim

**Affiliations:** 1 Department of Biotechnology, College of Life Science and Biotechnology, Yonsei University, Seoul 03722, Republic of Korea; rkdgksmf_@naver.com; 2 College of Pharmacy and Research Institute for Drug Development, Pusan National University, Busan 46241, Republic of Korea; nebbia1127@gmail.com (E.-J.K.); ddahye0620@gmail.com (D.L.); 3 Department of Food Science and Biotechnology, Dongguk University, Goyangsi 10326, Republic of Korea; 4 Graduate Program in Bioindustrial Engineering, Yonsei University, Seoul 03722, Republic of Korea; junrim2577@naver.com

**Keywords:** *Curcuma xanthorrhiza* supercritical extract (CXSE), xanthorrhizol (XAN), particulate matter (PM), skin damage, aryl hydrocarbon receptor (AhR)

## Abstract

**Background:** Particulate matter (PM) is a major environmental pollutant that induces oxidative stress, inflammation, and extracellular matrix (ECM) degradation, leading to skin damage and accelerated aging. Xanthorrhizol (XAN), a bioactive compound derived from *Curcuma xanthorrhiza* Roxb., exhibits anti-inflammatory and antioxidative properties, making it a promising candidate for protecting against PM-induced skin damage. This study investigated the protective effects of XAN and *C. xanthorrhiza* supercritical extract (CXSE) on PM-exposed skin cells. **Methods:** A 3D-reconstructed skin model and HaCaT human keratinocytes were exposed to PM (100 µg/mL) with or without CXSE or XAN. Histological analysis, enzyme-linked immunosorbent assay (ELISA), Western blot, reverse transcription-polymerase chain reaction (RT-PCR), and reporter gene assays were performed to assess the ECM integrity, inflammatory cytokine production, aryl hydrocarbon receptor (AhR) activation, and oxidative stress responses. **Results:** PM exposure activates AhR and mitogen-activated protein kinases (MAPK) signaling, increases reactive oxygen species (ROS) levels, and upregulates matrix metalloproteinases (MMPs) and inflammatory cytokines. CXSE and XAN suppresses AhR-mediated transcriptional activity and downregulates the expression of AhR target genes. Additionally, CXSE and XAN reduces ROS production by upregulating antioxidant enzyme-related genes. **Conclusions:** CXSE and XAN protect against PM-induced skin damage by inhibiting oxidative stress, inflammation, and ECM degradation, highlighting their potential as natural anti-pollution skincare ingredients.

## 1. Introduction

**Figure 1:**
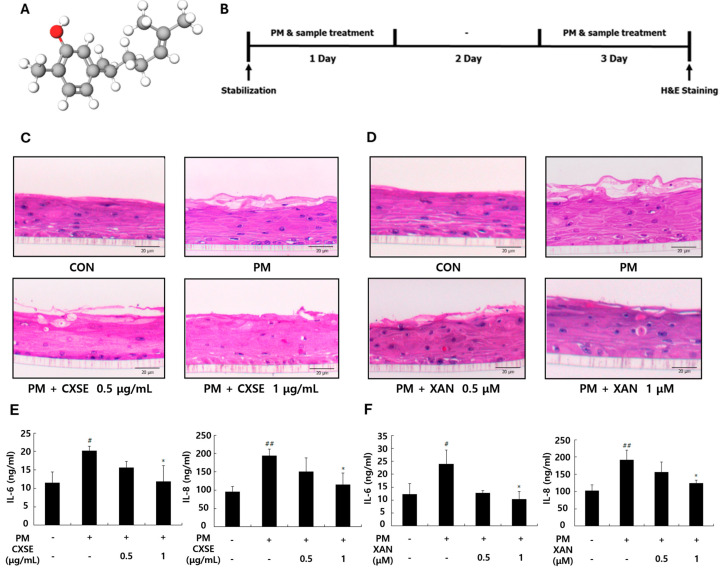
Effects of CXSE and XAN on the histological changes and pro-inflammatory cytokine production in a PM-stimulated, 3D-reconstructed human skin model. (**A**) The chemical structure of XAN. (**B**) Schematic overview of the experimental design. The 3D-reconstructed human skin tissues were treated with CXSE or XAN following PM exposure. (**C**,**D**) Histological changes in the epidermis after treatment with CXSE (**C**) and XAN (**D**) in the presence of 100 µg/mL of PM were assessed by H&E staining (magnification, ×100; scale bar, 20 μm). (**E**,**F**) Production of IL-6 and IL-8 following treatment with CXSE (**E**) or XAN (**F**) after PM stimulation, as measured by ELISA. All treatment with CXSE and XAN were performed after exposure to 100 µg/mL of PM for 72 h. Data are presented as the mean ± SD (% of untreated control) from three individual experiments. Statistical analysis was performed using one-way ANOVA followed by Duncan’s test. ^#^ *p* < 0.5 and ^##^ *p* < 0.01 vs. untreated control, and * *p* < 0.05 vs. PM-treated control. CXSE and XAN were used at concentrations of 0.5 and 1 µg/mL or µM, respectively. Symbols “-” in the figure indicate no treatment, and symbols “+” in the figure indicate treatment with PM.

*Curcuma xanthorrhiza* Roxb., or Java turmeric, has been widely used in traditional medicine due to its broad spectrum of biological activities [1]. Among its bioactive compounds, XAN (Figure 1A) is notable for its anti-inflammatory, antioxidant, antibacterial, neuroprotective, and hepatoprotective properties [1,2,3,4]. Previous studies have shown that XAN can effectively modulate key inflammatory pathways by reducing the phosphorylation of MAPKs and suppressing pro-inflammatory cytokines, such as IL-6 and IL-8, in various cell types [5,6]. These findings suggest that XAN may attenuate oxidative stress and inflammation, highlighting its potential for therapeutic applications in skincare.

Particulate matter (PM), a complex mixture of airborne pollutants, is a major environmental concern due to its adverse health effects that induce oxidative stress and inflammation in biological systems [7,8,9,10,11]. Notably, both the incidence and mortality rates of diseases associated with PM exposure are increasing [12,13]. Exposure to PM generates reactive oxygen species (ROS), which activate the MAPK signaling cascade and NF-κB, thereby subsequently upregulating matrix metalloproteinases (MMPs) and other inflammatory mediators [14,15,16]. This signaling cascade degrades extracellular matrix (ECM) proteins, such as collagen and elastin, thereby promoting skin inflammation and accelerating aging processes [17,18,19]. While much of the research on PM has focused on respiratory effects, its impact on the skin, the body’s outermost barrier, has emerged as an important target for intervention [20,21].

The skin, which is continuously exposed to environmental pollutants, is particularly vulnerable to PM-induced damage [20,22,23,24]. PM exposure is associated with increased oxidative stress, chronic inflammation, and the development of skin conditions, such as erythema, dryness, and wrinkles [25]. Recent studies have also linked PM exposure to chronic skin disorders, including atopic dermatitis, psoriasis, acne, and skin aging [26,27]. In addition, several natural products have been reported to reduce the harmful effects of PM exposure [28,29,30].

PM-induced oxidative stress causes lipid peroxidation, protein oxidation, and DNA damage, leading to impaired cellular function and the initiation of inflammatory signaling pathways, such as AhR, NF-κB, and MAPK [31,32,33]. Given the growing prevalence of air pollution and its detrimental effects on skin health, there is an urgent need for effective strategies to mitigate PM-induced damage. Recent studies have identified targeting ROS and MAPK signaling pathways as a promising approach to preserve skin integrity and function under environmental stress [19,34,35].

This study investigated the potential of XAN as a cosmetic ingredient to mitigate PM-induced skin damage. By leveraging its anti-inflammatory and antioxidant properties, XAN may reduce oxidative stress, suppress inflammatory signaling, and prevent ECM degradation in PM-exposed skin cells. Our findings are expected to support the application of XAN in anti-pollution skincare formulations and contribute to the development of protective strategies in dermatological and cosmetic research.

## 2. Results

### 2.1. CXSE and XAN Mitigate PM-Induced Histological Changes and Suppress Inflammatory Cytokines in 3D-Reconstructed Skin

Figure 1A demonstrates the three-dimensional structure of XAN. Prior to evaluating the protective effects of CXSE and XAN against PM-induced damage, we confirmed their cytocompatibility. MTT assay revealed that CXSE and XAN did not exhibit cytotoxicity and slightly enhanced cell proliferation at concentrations of up to 1 µg/mL or 1 µM, respectively (Figure S1). We investigated the protective effects of CXSE and XAN against PM-induced damage using a 3D-reconstructed skin model. Figure 1B presents a schematic overview of the experimental design. H&E staining revealed the histological structure and morphology of the stratum corneum. The PM-treated group exhibited significant damage in the stratum corneum compared to the control group. However, treatment with CXSE and XAN alleviated the morphological disruptions in the epidermis layer, with a more pronounced effect observed at the higher concentration (Figure 1C,D).

To assess inflammatory responses, we measured the secretion of cytokines in 3D-reconstructed skin tissue. PM treatment was found to significantly increase the production of IL-6 and IL-8, whereas treatment with CXSE and XAN effectively reduced the levels of these cytokines (Figure 1E,F). These findings suggest that CXSE and XAN may help protect against PM-induced skin damage.

### 2.2. CXSE and XAN Modulate the Expression of AhR and Attenuate Oxidative Responses After PM Exposure

**Figure 2:**
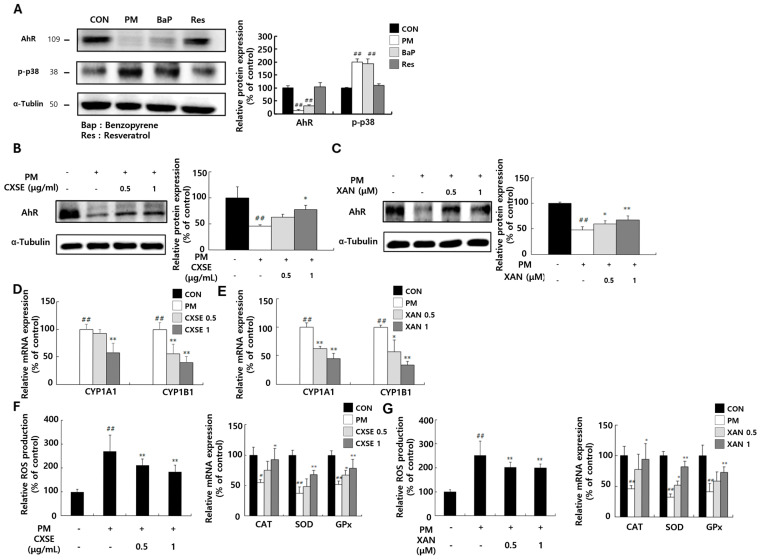
Inhibitory effects of CXSE and XAN on the expression of AhR and cytochrome P450 enzymes and the production of ROS in HaCaT cells. (**A**) Protein expression of AhR and phosphorylated p38 in cells treated with PM, benzo[a]pyrene (BaP), and resveratrol (Res). (**B**,**C**) Effect of CXSE (**B**) or XAN (**C**) on AhR protein expression following PM stimulation. (**D**,**E**) Relative mRNA expression of *CYP1A1* and *CYP1B1* in PM-exposed cells treated with CXSE (**D**) or XAN (**E**), as measured by RT-PCR. (**F**,**G**) The intracellular ROS levels and mRNA expression of antioxidant enzymes (CAT, SOD, and GPx) in PM-stimulated cells treated with CXSE (**F**) or XAN (**G**). All data are expressed as the mean ± SD (% of untreated control) from three independent experiments. Statistical significance was determined using one-way ANOVA followed by Duncan’s multiple range test. ^#^ *p* < 0.5 and ^##^ *p* < 0.01 vs. untreated control, and * *p* < 0.05 and ** *p* < 0.01 vs. PM-treated control. CXSE and XAN were used at 0.5 or 1 μg/mL or μM, respectively. Symbols “-” in the figure indicate no treatment, and symbols “+” in the figure indicate treatment with PM.

As shown in Figure 2A, a reduction in the expression of AhR was observed following treatment with PM and benzopyrene (BaP), a major polycyclic aromatic hydrocarbon (PAH) and a component of PM. This finding suggests that these environmental toxins suppress the level of AhR or promote its degradation. In contrast, AhR expression in the resveratrol (Res)-treated group remained comparable to that in the control group, indicating that Res may prevent the suppression of AhR or exert a protective effect. Additionally, the elevated phospho-p38 levels in the PM- and BaP-treated groups highlighted the activation of stress or inflammatory pathways in response to environmental stress, further supporting the role of the p38 MAPK pathway in stress signaling.

To further investigate the impact of PM on the AhR-mediated XRE binding activity, we used COS-7 cells, which are known for their high transfection efficiency (Figure S2A). In the Bap-treated groups (Figure S2B) and PM-treated groups (Figure S2C), CXSE and XAN were administered at two different concentrations to evaluate their inhibitory effects on AhR-mediated XRE binding activity. In HaCaT cells, PM exposure resulted in a reduction in AhR protein levels. However, treatment with CXSE following PM exposure restored the expression of AhR protein in a concentration-dependent manner (Figure 2B,C). PM binding to AhR led to its nuclear translocation and subsequent upregulation of the target gene CYP. However, when CXSE was administered at varying concentrations, a significant reduction in PM-induced CYP mRNA expression was observed (Figure 2D). A similar pattern was observed in the group treated with XAN (Figure 2E). These findings suggest that CXSE and XAN suppress PAH-induced oxidative stress by modulating AhR signaling.

Next, ROS production was examined in PM-treated HaCaT keratinocyte cells to evaluate the inhibitory effects of CXSE and XAN on PM-induced oxidative stress. Compared to the control group, the PM-treated group showed increased ROS production. However, treatment with CXSE and XAN significantly reduced ROS levels (Figure 2F). ROS induces oxidative stress by damaging cellular components, including DNA, proteins, and lipids [36]. To counteract this damage, cells utilize various antioxidant enzymes, among which catalase (CAT), superoxide dismutase (SOD), and glutathione peroxidase (GPx) play critical roles in antioxidant defense [37]. To investigate how cells respond to ROS and regulate antioxidant defense mechanisms under oxidative stress, we evaluated the mRNA expression levels of CAT, SOD, and GPx. The expression levels of the three antioxidant enzymes were effectively restored in the CXSE and XAN-treated groups at both concentrations following PM exposure when compared to the group exposed to PM alone. Notably, CXSE and XAN significantly restored *catalase* mRNA expression, suggesting a targeted regulatory effect (Figure 2G). These findings indicate that CXSE and XAN enhance endogenous antioxidant defenses and protect cells from PM-induced oxidative damage.

### 2.3. CXSE and XAN Downregulate MAPK Pathway and Suppress MMP mRNA Expression in PM-Treated HaCaT Cells

The MAPK signaling pathway is likely involved in mediating inflammation and regulating *MMP-1, MMP-2*, and *MMP-13* expression. According to previous reports, MAPK signaling upregulates the expression of MMPs, which degrade ECM proteins, such as collagen, elastin, and proteoglycans [19]. MMP-mediated degradation of the ECM proteins compromises the structural integrity of the skin, eventually leading to wrinkle formation [38].

**Figure 3:**
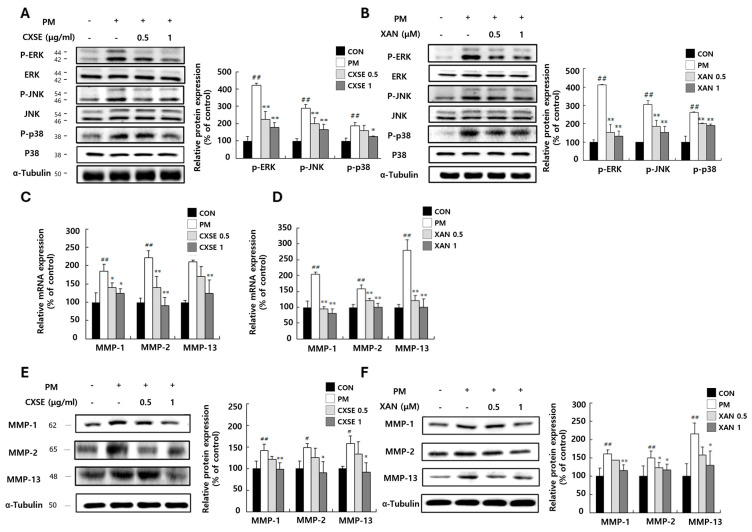
The inhibitory effects of CXSE and XAN on the MAPK pathway and MMP expression in HaCaT cells. (**A**,**B**) Phosphorylation levels of MAPK proteins (p-ERK, p-JNK, and p-p38) in PM-stimulated cells treated with CXSE (**A**) or XAN (**B**), as assessed by Western blotting. (**C**,**D**) The relative mRNA expression of *MMP-1, MMP-2*, and *MMP-13* in PM-treated cells following treatment with CXSE (**C**) or XAN (**D**), as measured by RT-PCR. (**E**,**F**) Protein expression of MMP-1 in CXSE-treated (**E**) or XAN-treated (**F**) cells after PM stimulation. Data are expressed as the mean ± SD (% control) from three individual experiments. Group differences were assessed using one-way analysis of variance (ANOVA) followed by Duncan’s test. ^#^ *p* < 0.5 and ^##^ *p* < 0.01 vs. untreated control, and * *p* < 0.05 and ** *p* < 0.01 vs. PM-treated control. All data are expressed as the mean ± SD (% of untreated control) from three independent experiments. Statistical significance was determined using one-way ANOVA followed by Duncan’s multiple range test. ^##^ *p* < 0.01 vs. untreated control, and * *p* < 0.05 and ** *p* < 0.01 vs. PM-treated control. CXSE and XAN were used at 0.5 or 1 μg/mL or μM, respectively. Symbols “-” in the figure indicate no treatment, and symbols “+” in the figure indicate treatment with PM.

To determine whether the MAPK signaling pathway contributes to regulating MMP expression at the protein level, we examined the phosphorylation of ERK, JNK, and p38. PM treatment significantly increased the phosphorylation of these kinases, indicating activation of the MAPK pathway. However, CXSE treatment markedly reduced their phosphorylation levels (Figure 3A). A similar reduction was observed in the XAN-treated group (Figure 3B), suggesting that both CXSE and XAN may inhibit MAPK pathway activation. Furthermore, we investigated the effects of CXSE and XAN on PM-induced *MMP-1, MMP-2*, and *MMP-13* expressions. In the PM-treated control group, the expression of MMPs was significantly upregulated, suggesting that PM-induced inflammation contributed to this increase. However, treatment with high concentrations of CXSE and XAN markedly reduced the expression of MMPs at both the mRNA and protein levels in a dose-dependent manner (Figure 3C–F). These findings confirm that PM exposure activates the MAPK signaling pathway, leading to increased MMP expression, while CXSE and XAN effectively counteract this response.

### 2.4. CXSE and XAN Reduce Inflammatory Responses in PM-Treated Keratinocytes

Previous studies have reported that MAPK activation triggers NF-κB, a key transcription factor that promotes the production of inflammatory cytokines, such as IL-6 and IL-8, thereby contributing to inflammatory responses characterized by erythema and itching [34].

**Figure 4:**
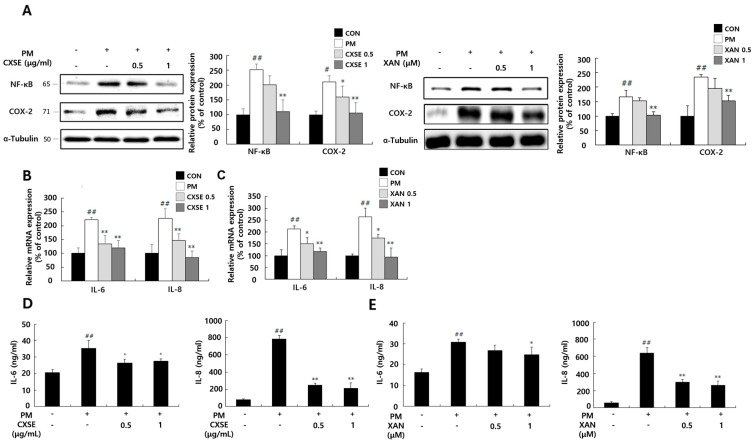
Inhibitory effects of CXSE and XAN on PM-induced inflammation in HaCaT cells. HaCaT cells were exposed to 100 µg/mL of PM for 24 h and subsequently treated with CXSE or XAN. (**A**) Protein expression of NF-κB and COX-2 was evaluated by Western blotting using α-tubulin as a loading control. (**B**,**C**) mRNA expression level of *IL-6* and *IL-8* were determined by RT-PCR after treatment with CXSE (**B**) or XAN (**C**). (**D**,**E**) IL-6 and IL-8 concentration was determined on the PM-treated cells following treatment with CXSE (**D**) or XAN (**E**). All data are expressed as the mean ± SD (% of untreated control) from three independent experiments. Statistical significance was determined using one-way ANOVA followed by Duncan’s multiple range test. ^#^ *p* < 0.5 and ^##^ *p* < 0.01 vs. untreated control, and * *p* < 0.05 and ** *p* < 0.01 vs. PM-treated control. CXSE and XAN were used at 0.5 or 1 μg/mL or μM, respectively. Symbols “-” in the figure indicate no treatment, and symbols “+” in the figure indicate treatment with PM.

To evaluate the anti-inflammatory effects of CXSE and XAN, we examined the mRNA and protein expression levels, as well as the production of cytokines. We examined the expression patterns of two inflammatory factors, including NF-κB and COX-2, which regulate and propagate the inflammatory response (Figure 4A,B). PM treatment increased the mRNA expression of *IL-6* and *IL-8*, whereas their expression levels were reduced in the CXSE- and XAN-treated groups (Figure 4C,D). This trend was also confirmed at the protein level, with reduced cytokine production in the CXSE- and XAN-treated groups (Figure 4E). These results suggest that CXSE and XAN effectively mitigate PM-induced inflammatory responses by downregulating pro-inflammatory cytokines. This highlights the potential of CXSE and XAN as therapeutic agents for preventing or alleviating PM-induced skin inflammation and oxidative stress-related conditions.

## 3. Discussion

This study demonstrated the protective effects of CXSE and its major component, XAN, against PM-induced skin damage, oxidative stress, inflammation, and ECM degradation. PM exposure activated the MAPK signaling pathway, increased MMP expression, and elevated ROS levels, leading to histological damage and heightened cytokine production. However, CXSE and XAN inhibited MAPK activation, downregulated MMPs, restored the expression of antioxidant enzyme, and reduced IL-6 and IL-8 levels. Additionally, they prevented the PM-induced suppression of AhR, suggesting a role in maintaining skin homeostasis. Our results provide mechanistic insight into how CXSE and XAN modulate key signaling pathways and support their potential as active ingredients in anti-pollution skincare formulations.

The skin serves as the primary barrier against environmental pollutants, such as PM, which cause oxidative stress, inflammation, and degradation of ECM [22]. Toxic constituents of PM, such as PAHs including fluoranthene, pyrene, and benzo[a]pyrene, activate AhR, which stimulates the cytochrome P450 enzyme family and subsequently increases ROS generation and inflammatory responses [39,40].

A key finding of this study was the potent antioxidant activity exhibited by CXSE and XAN. PM exposure increased intracellular ROS production, which contributes significantly to oxidative skin damage. CXSE and XAN markedly reduced ROS levels and restored antioxidant enzyme expressions, including catalase, SOD, and GPx, indicating a robust protective mechanism against oxidative stress.

AhR signaling represents another critical pathway implicated in PM-induced skin damage. PM exposure results in enhanced nuclear translocation of AhR and the upregulated expression of *CYP1A1*, which is a hallmark of PAH metabolism [23]. Treatment with CXSE and XAN prevents AhR nuclear translocation, thus reducing downstream *CYP1A1* gene expression. These results suggest that CXSE and XAN interfere with the binding of PAHs to AhR, modulating the signaling cascade crucial for reducing oxidative damage and inflammation.

Moreover, PM-induced ROS generation also activates the MAPK signaling pathway, a critical mediator of inflammatory and stress responses that triggers NF-κB activation and promotes ECM degradation through enhanced MMP expression [34,41,42]. Our results show that CXSE and XAN effectively suppress MAPK activation, inhibit NF-κB activation, and reduce the expression of *MMP-1, MMP-2*, and *MMP-13*. These effects collectively indicate a powerful anti-inflammatory response and the preservation of ECM integrity.

Inflammatory mediators, notably IL-6 and IL-8, were significantly elevated upon PM exposure. CXSE and XAN effectively reduced the secretion of these cytokines, which is consistent with earlier observations demonstrating XAN’s inhibitory effect on pro-inflammatory cytokines, such as IL-6, IL-1β, and TNF-α, in RAW 264.7 cells [6], as well as CXSE’s cytokine reduction in obese mice [43]. Thus, CXSE and XAN exhibit potent anti-inflammatory properties by targeting the upstream signaling pathways involved in cytokine production.

In terms of clinical application, these findings strongly suggest the utility of CXSE and XAN in skincare formulations aimed at mitigating PM-induced premature skin aging, irritation, and collagen degradation. The preservation of collagen integrity through MMP inhibition further underscores the therapeutic potential of these compounds in maintaining skin elasticity and preventing wrinkle formation [38].

While our results provide substantial in vitro evidence supporting the protective effects of CXSE and XAN against PM-induced skin damage, some limitations exist. The complexity of human skin responses might not be fully captured by our in vitro models. Therefore, future studies involving in vivo validation or clinical trials are crucial to confirm the efficacy, bioavailability, and long-term safety of these compounds. Additionally, further research is required to elucidate the precise molecular interactions of CXSE and XAN with AhR and MAPK pathways using targeted inhibition approaches. Finally, formulation stability and potential cytotoxicity assessments should be conducted to optimize their practical application in commercial skincare products.

## 4. Materials and Methods

### 4.1. Preparation of CXSE and XAN

CXSE was prepared following previously established methods [44]. Briefly, C. xanthorrhiza rhizomes were obtained from Cosmax NBT (Seoul, Republic of Korea) and extracted using a supercritical CO_2_ fluid extraction system (SCFE-P400, Ilshin Autoclave Co., Ltd., Daejeon, Republic of Korea) [45]. The ground rhizomes were loaded into a high-pressure vessel maintained at 50 °C. CO_2_ was introduced at 3.2 L/min and 40 MPa. The extraction yielded CXSE at 8.0% (*w*/*w*) [46,47], with XAN comprising 30.0% (*w*/*w*) of the extract. XAN was further isolated as previously described [43].

### 4.2. Preparation of Fine Particulate Matter Samples

Standard Reference Material (SRM 2786, NIST, Gaithersburg, MD, USA) with a mean particle diameter less than 4 μm was used as the PM source [48]. SRM2786 was suspended in Dulbecco’s phosphate-buffered saline (DPBS; Dynebio, Gyeonggi, Republic of Korea) at 10 mg/mL and sonicated for 1 h before treatment to prevent particle aggregation.

### 4.3. Cell Culture

HaCaT (human keratinocytes) and COS-7 (monkey kidney fibroblast) cells were purchased from the American Type Culture Collection (ATCC; Manassas, VA, USA). Cells were cultured in DMEM (Hyclone Laboratories, Logan, UT, USA) containing 10% fetal bovine serum (FBS; Hyclone), 1% penicillin, and 1% streptomycin (Invitrogen, Carlsbad, CA, USA) at 37 °C in a humidified 5% CO_2_ incubator. For the experiments, cells were seeded and treated with CXSE or XAN in the presence of 100 µg/mL of PM.

### 4.4. AhR Reporter Gene Luciferase Assay

COS-7 cells were transfected with pGL4.43[luc2P/XRE/Hygro] reporter vector (Promega, Madison, WI, USA) using Plus reagent and Lipofector (AptaBio, Gyeonggi, Republic of Korea). After 4 h, cells were incubated in DMEM with 10% FBS for 24 h. Cells were then treated with CXSE or XAN in the presence of 100 µg/mL of PM for 24 h, which were lysed with NP-40 buffer (Elpis Biotech, Daejeon, Republic of Korea), and luciferase activity was measured using a MicroLumatPlus luminometer (Berthold, Wildbad, Germany).

### 4.5. Reverse Transcription-Polymerase Chain Reaction (RT-PCR)

Total RNA was isolated from HaCaT cells using TRIzol reagent (Takara, Shiga, Japan). RNA concentration was quantified spectrophotometrically with a NanoDrop 1000 spectrophotometer (Thermo Fisher Scientific, Waltham, MA, USA). cDNA was synthesized using reverse transcriptase premix (Elpis Biotech) according to the standard protocol. The cDNA was amplified with Taq PCR PreMix (CellSafe, Gyeonggi, Republic of Korea) and specific primers (Bioneer, Daejeon, Republic of Korea), as listed in Table 1. PCR products were resolved on 1.5% agarose gel and visualized using a G: BOX EF imaging system (Syngene, Cambridge, UK) and the GeneSys 1.8.0 software program.

### 4.6. Western Blot Analysis

Cells were lysed with NP-40 buffer (Elpis Biotech) containing protease inhibitors (Sigma-Aldrich, St. Louis, MO, USA). Protein concentration was measured using the Bradford assay (Bio-Rad, Hercules, CA, USA). Equal amounts of protein were separated by SDS-PAGE and transferred to nitrocellulose membranes (GE Healthcare, Piscataway, NJ, USA). Membranes were blocked with 5% skim milk and probed overnight with primary antibodies (1:1000) against NF-κB; AhR (Santa Cruz Biotechnology, Santa Cruz, CA, USA); tumor protein p53 (p53); cytochrome c; cyclooxygenase-2 (COX-2); p-c-Jun N-terminal kinase (p-JNK); p-extracellular signal-regulated kinase (p-ERK); p-p38; ERK; JNK; p38; α-tubulin (Cell Signaling, Beverly, MA, USA); and MMP-1 (Bioworld, Dublin, OH, USA). After incubation with HRP-conjugated secondary antibodies (Bethyl Laboratories, Inc., Montgomery, TX, USA), signals were detected using ECL solution (Amersham Biosciences, Little Chalfont, UK) and captured with a G: BOX EF imaging system (Syngene).

### 4.7. Measurement of ROS

ROS production was measured using 2,7-dichlorodihydrofluorescein diacetate (H_2_DCFDA; Molecular Probes, Eugene, OR, USA). Cells were treated with CXSE or XAN and 100 µg/mL of PM for 24 h. After staining with 10 µM of H_2_DCFDA for 30 min, cells were lysed and centrifuged. Supernatants were analyzed at 485 nm excitation and 530 nm emission using a SpectraMax Gemini EM reader (Molecular Devices, Sunnyvale, CA, USA).

### 4.8. 3D-Reconstructed Human Skin Model Culture

Neoderm-E (Tegoscience, Seoul, Republic of Korea) was used as a 3D-reconstructed human skin model. Tissues were maintained in a maintenance medium (Tegoscience) at 37 °C in a humidified atmosphere containing 5% CO_2_. The skin tissues were treated daily with CXSE or XAN in the presence of 100 µg/mL of PM for three consecutive days (with daily medium changes).

### 4.9. Histological Analysis

Tissues were fixed in 10% formalin (Junsei Chemical Co., Ltd., Tokyo, Japan), embedded in paraffin, and then sectioned and stained with hematoxylin and eosin (H&E). Sections were observed under a light microscope (CK40; Olympus, Tokyo, Japan) equipped with an eXcope T500 camera (DIXI Science, Daejeon, Republic of Korea).

### 4.10. Enzyme-Linked Immunosorbent Assay (ELISA)

Concentrations of IL-6 and IL-8 in a conditioned medium from HaCaT cells and 3D-reconstructed human skin tissues were measured using commercial human ELISA kits (R&D Systems, Minneapolis, MN, USA). Absorbance was measured at 450 nm using a VersaMax microplate reader (Molecular Devices).

### 4.11. Statistical Analysis

All experiments were performed in triplicate, and the results were expressed as the mean ± standard deviation (SD). Statistical analyses were conducted using SPSS version 25.0 (IBM Corp., Chicago, IL, USA). Group differences were evaluated by one-way analysis of variance (ANOVA) followed by Duncan’s post hoc test. * *p* < 0.05, ** *p* < 0.01, ^#^ *p* < 0.05, and ^##^ *p* < 0.01.

## 5. Conclusions

In this study, we demonstrated that CXSE and its major component, XAN, effectively protect skin cells from PM-induced oxidative stress, inflammation, and ECM degradation. CXSE and XAN reduced ROS production, restored antioxidant enzyme expression, and inhibited the activation of AhR and MAPK signaling pathways. These treatments also downregulated pro-inflammatory mediators and MMPs, preserving the structural and functional integrity of the skin. The protective effects were confirmed in both HaCaT cells and a 3D-reconstructed human skin model, highlighting their relevance for topical application. Taken together, our findings support the potential use of CXSE and XAN as bioactive ingredients in anti-pollution skincare products aimed at preventing or mitigating PM-induced skin damage.

## Figures and Tables

**Table 1:** The primer sequences used in reverse transcription-polymerase chain reaction (RT-PCR) analysis.

Gene	Direction	Sequence (5′-3′)
*CYP1A1*	Forward	CTACCCAACCCTTCCCTGAAT
	Reverse	CGCCCCTTGGGGATGTAAAA
*CYP1* *B1*	Forward	CTGCGACTCCAGTTGTGAGA
	Reverse	AAGGAACTGGGACCTTTGCC
*MMP-1*	Forward	AAGTCAAGTTTGTGGCTTAT
	Reverse	GACTCATGTCTCCTGTCTCT
*Catalase*	Forward	GCCACAGGAAAGTACCCCTC
	Reverse	CGGTGAGTGTCAGGATAGGC
*IL-6*	Forward	ATGAGGAGACTTGCCTGGTG
	Reverse	ACAACAATCTGAGGTGCCCA
*IL-8*	Forward	CCAGGAAGAAACCACCGGAA
	Reverse	CCTCTGCACCCAGTTTTCCT
*GAPDH*	Forward	CTCCTGTTCGACAGTCAGCC
	Reverse	TCGCCCCACTTGATTTTGGA

## Data Availability

The original contributions presented in this study are included in the article/Supplementary Material. Further inquiries can be directed to the corresponding authors.

## Supplementary Materials

The following supporting information can be downloaded at: https://www.mdpi.com/article/10.3390/ph18060808/s1, Figure S1: Cell viability of HaCaT cells treated with CXSE and XAN. HaCaT cells were treated with various concentrations of CXSE (µg/mL) and XAN (µM) for 24 h. Cell viability was assessed using the MTT assay. Figure S2: CXSE and XAN inhibited the AhR transcription in PM-treated COS-7 cells. (A) Effect of PM on AhR-mediated XRE binding activity. COS-7 cells were transfected with pGL4.43[luc2P/XRE/Hygro] vector then treated with 12.5, 25, 50, and 100 µg/mL of PM for 24 h. (B) The inhibitory effect of CXSE and XAN on the AhR-mediated XRE binding activity in the BaP-treated group. (C) The inhibitory effect of CXSE and XAN on the AhR-mediated XRE binding activity in the PM-treated group. The pGL4.43[luc2P/XRE/Hygro] vector was then treated with CXSE or XAN in the presence of 100 µg/mL of PM for 24 h.

## Abbreviations

CXSE	*Curcuma xanthorrhiza* supercritical extract
XAN	Xanthorrhizol
PM	Particulate matter
AhR	Aryl hydrocarbon receptor
ECM	Extracellular matrix
Res	Resveratrol
BaP	Benzo[a]pyrene
CAT	Catalase
SOD	Superoxide dismutase
GPx	Glutathione peroxidase
MMPs	Matrix metalloproteinases
ROS	Reactive oxygen species
PAHsMAPK	Polycyclic aromatic hydrocarbonsMitogen-activated protein kinases
