# Supplementary material for: Protective Effects of Xanthorrhizol-Rich Extracts Against PM-Induced Skin Damage in Human Keratinocytes and 3D-Reconstructed Skin Models
Source: Pharmaceuticals (Basel). 2025 May 28;18(6):808. doi: 10.3390/ph18060808 (PMC12196211; doi:10.3390/ph18060808)
Supplement: Supplementary file 1 [file pharmaceuticals-18-00808-s001.zip › pharmaceuticals-3555998-supplementary.pdf]

Supplementary Material

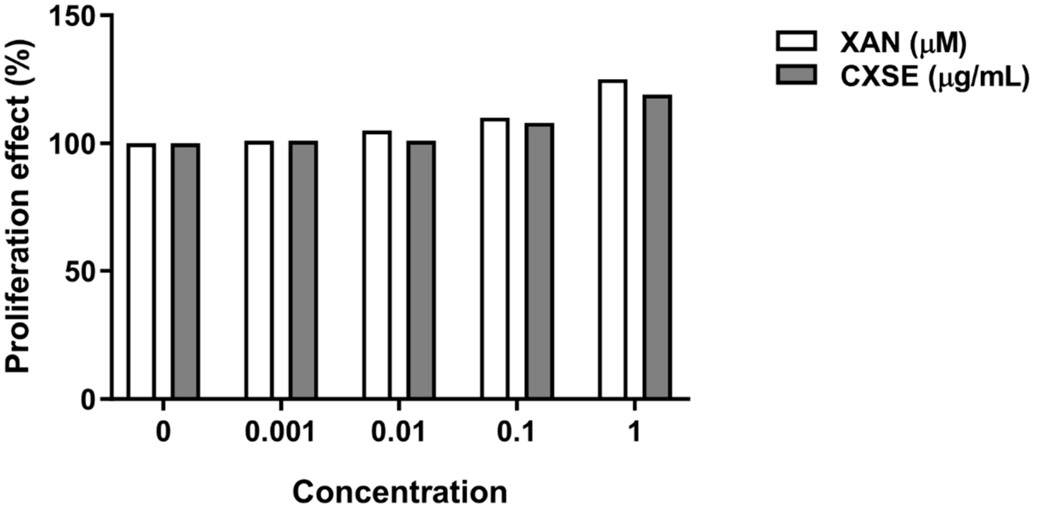

Figure S1: Cell viability of HaCaT cells treated with CXSE and XAN. HaCaT cells were treated with various concentrations of CXSE (1 μg/mL) and XAN (1 μM) for 24 h. Cell viability was assessed using the MTT assay.

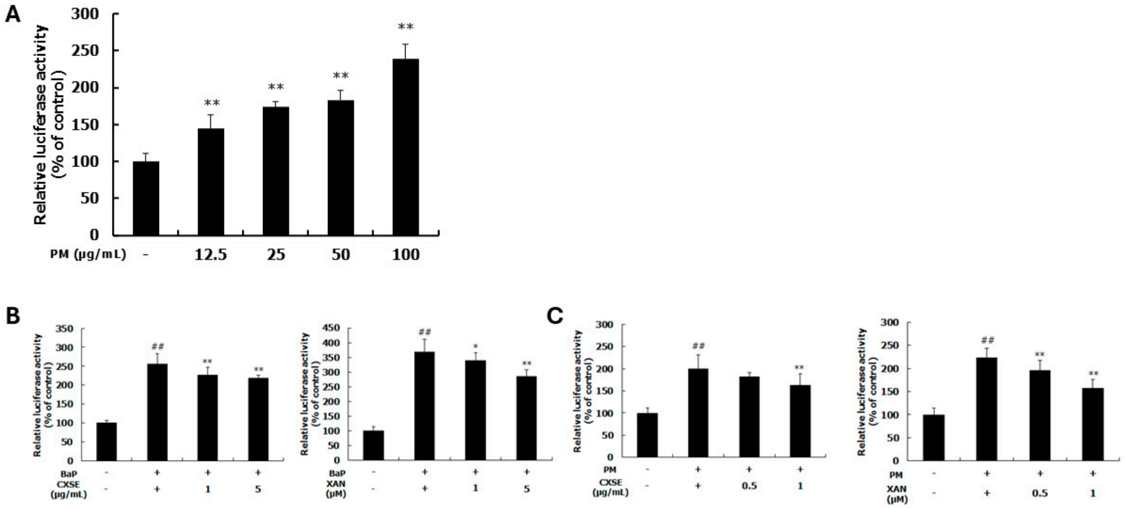

Figure S2: CXSE and XAN inhibit AhR transcription in PM-treated COS-7 cells (A) Effect of PM on AhR-mediated XRE binding activity. COS-7 cells were transfected with pGL4.43[luc2P/XRE/Hygro] vector then treated with 12.5, 25, 50 and 100 μg/mL PM for 24 h. (B) Inhibitory Effect of CXSE and XAN on AhR-Mediated XRE Binding Activity in the BaP-Treated Group. (C) Inhibitory Effect of CXSE and XAN on AhR-Mediated XRE Binding Activity in the PM-Treated Group. pGL4.43[luc2P/XRE/Hygro] vector then treated with CXSE or XAN in the presence of 100 μg/mL PM for 24 h.
